# Supplementary material for: A V0 core neuronal circuit for inspiration
Source: Nat Commun. 2017 Sep 15;8:544. doi: 10.1038/s41467-017-00589-2 (PMC5601429; doi:10.1038/s41467-017-00589-2)
Supplement: Supplementary file 1 — Supplementary Information [file 41467_2017_589_MOESM1_ESM.pdf]

Description of Supplementary Files

File Name: Supplementary Information
Description: Supplementary Tables, Supplementary Figures

File Name: Supplementary Movie 1
Description: Left-right synchronized breathing of a wildtype pup at P0 and left-right desynchronized breathing of a $Dbx1^{cre}$; $Robo3^{lox/lox}$ mutant pup at P0 (for each sequence, duration: 6s, speed: x0.5).

File Name: Peer Review File

Supplementary Table 1. Bilateral distribution of phrenic premotor neurons.

Premotor region	Ipsi-lateral	Contra-lateral	p-values	
**rVRG**	35.66 ± 1.55	34.94 ± 2.10	0,7819	ns
**PB/KF**	7.54 ± 0.60	1.70 ± 0.26	0,0002	***
**preBötC**	3.09 ± 0.35	1.98 ± 0.42	0,1050	ns
**BötC**	2.06 ± 0.28	1.40 ± 0.24	0,0591	ns
**NTS**	2.16 ± 0.64	0.49 ± 0.27	0,0116	*
**LPGi**	1.80 ± 0.57	0.73 ± 0.29	0,0532	ns
**Ve**	0.80 ± 0.24	1.13 ± 0.34	0,3118	ns
**Gi**	1.51 ± 0.31	0.41 ± 0.16	0,0206	*
**IRt**	1.08 ± 0.56	0.68 ± 0.30	0,2414	ns
**Raphe**	0.83 ± 0.46			

Average number of Ph-preMNs (expressed as percent of the total number of trace$^+$ cells, n=6 experiments) in premotor regions ipsi-lateral and contra-lateral to the injected hemi-diaphragm. Note the large predominance of premotor regions hosting even numbers of neurons on both sides of the midline. A significant (t-test) ipsilateral projection bias is only attested in the PB/KF, the NTS and the Gi that together represent less than 15% of all Ph-preMNs.

Supplementary Figure 1

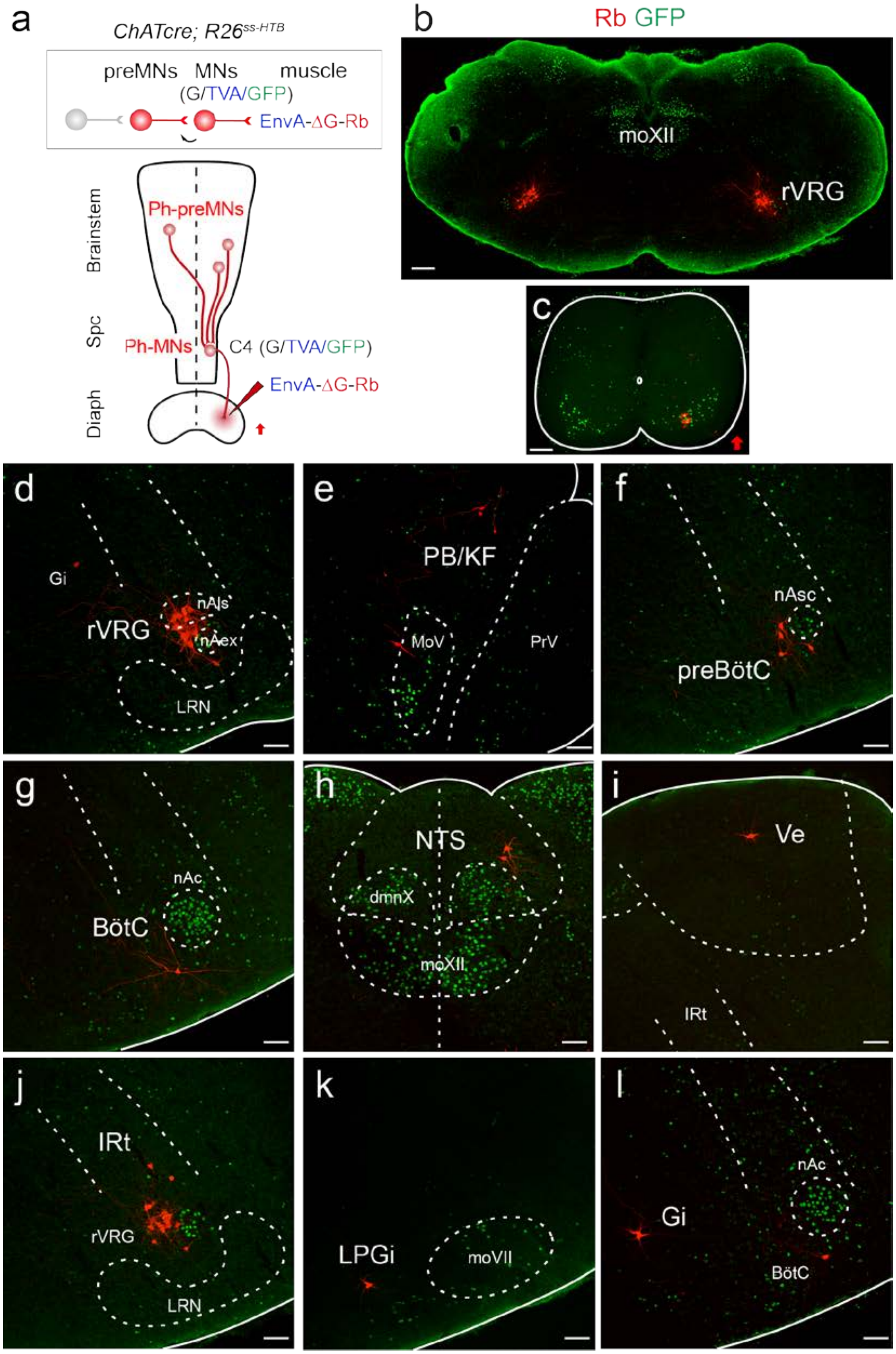

Supplementary Figure 1. Monosynaptic tracing from the Ph-MNs in *ChATcre; R26*^{ssHTB} in the neonatal mouse at P9. **a**, Tracing scheme for exclusive transsynaptic spread from motor neurons following unilateral injections of the diaphragm with EnvA-ΔG-Rb viral vector. In the *ChATcre;R26*^{ssHTB} background, MNs express transgenes encoding the envelop protein receptor TVA required for infection by EnvA pseudotyped viral vector, G for complementation and fluorescent protein GFP for labeling. **b**,**c** Brainstem transverse sections showing respectively, bilaterally distributed trace^{+} Ph-preMNs of the rVRG (b) and ipsilaterally located seeding trace^{+} Ph-MNs. **d-l,** Representative images of transverse sections of the brainstem showing trace^{+} Ph-preMNs in the rVRG (**d**), in the PB/KF (**e**), in the preBötC area (**f**), in the BötC (**g**), in the NTS (**h**), in Ve nuclei (**i**), in the lRt (**j**), in the LPGi (**k**) and Gi (**l**). Scale bars: b,c ,200µm; d-l,100µm.

Supplementary Figure 2

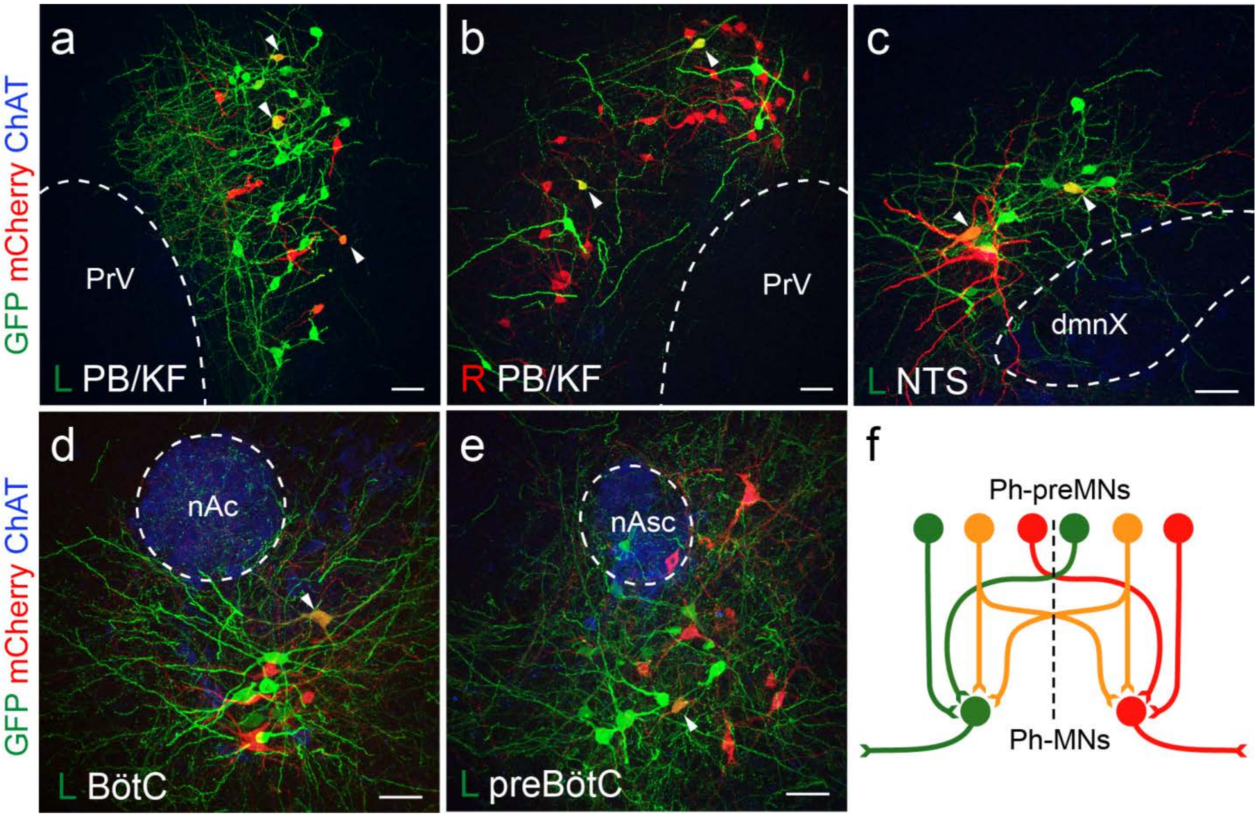

Supplementary Figure 2. Individual Ph-preMNs in locations outside the rVRG also project bilaterally on Ph-MNs. Red and green Rb respectively injected in the right and left diaphragm result in double labeled yellow Ph-preMNs (arrowheads). **a**, The left PB/KF (green l to indicate the color of the ipsi-laterally injected Rb) bears an excess of green vs red trace^{+} cells. **b**, Inverse proportions of green and red trace^{+} cells in the right PB/KF. **c**, Same for the left NTS. **d**, Same for the left BötC. **e**, Same for the left preBötC. Note that double labeled Ph-preMNs are present in these premotor regions irrespective their ipsilaterally biased (PB/KF, NTS) or not (BötC, preBötC) descending projections. **f**, Schematic of labeling and projections profiles of Ph-preMNs. Scale bars: 50µm.

Supplementary Figure 3

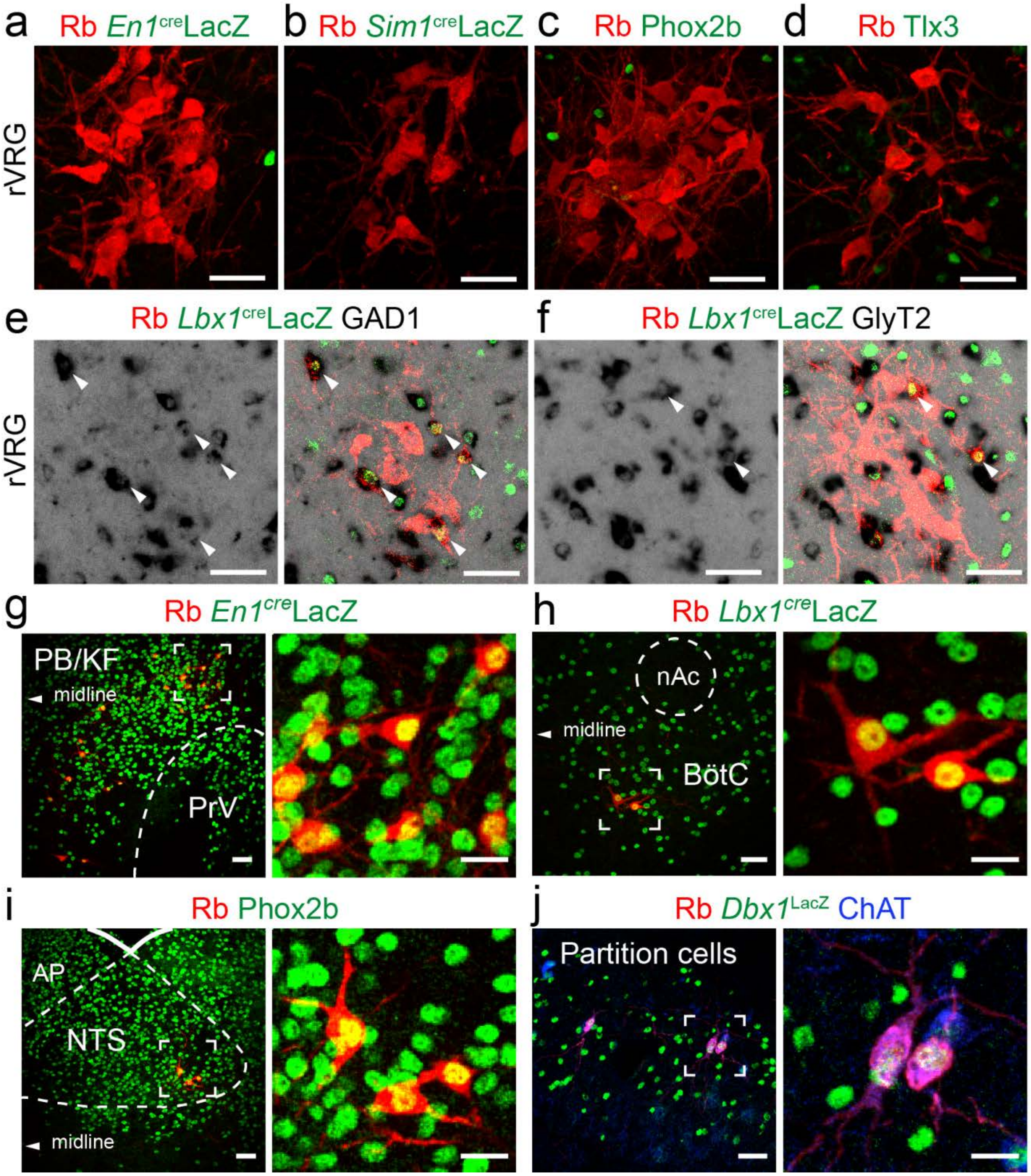

Supplementary Figure 3. Identities of the Ph-preMNs. **a-d**, Transverse sections showing that trace^{+} rVRG neurons have neither histories of expression of En1 (**a**) nor of Sim1 (**b**), and express neither Phox2b (**c**) nor Tlx3 (**d**). **e,f** combined IHC and ISH in *Lbx1cre;LacZ* mice showing that dB rVRG trace^{+} neurons express GAD1 (**e**) and GlyT2 (**f**). **g**, Traced Ph-preMNs in the PB/KF derive from En1-expressing progenitors shown at low magnification (left) and in the zoomed inset (right). **h**, Traced Ph-preMNs in the BötC derive from Lbx1-expressing precursors shown at low magnification (left) and in the zoomed inset (right). **i**, Traced Ph-preMNs in the NTS express Phox2b shown at low magnification (left) and in the zoomed inset (right). **j**, Sagittal section showing that traced partition cells in the cervical spinal cord are cholinergic and derive from Dbx1-expressing progenitors shown at low magnification (left) and in the zoomed inset (right). Scale bars: a-f, 50µm; g-j left, 50µm; g-j right, 20µm.

Supplementary Figure 4

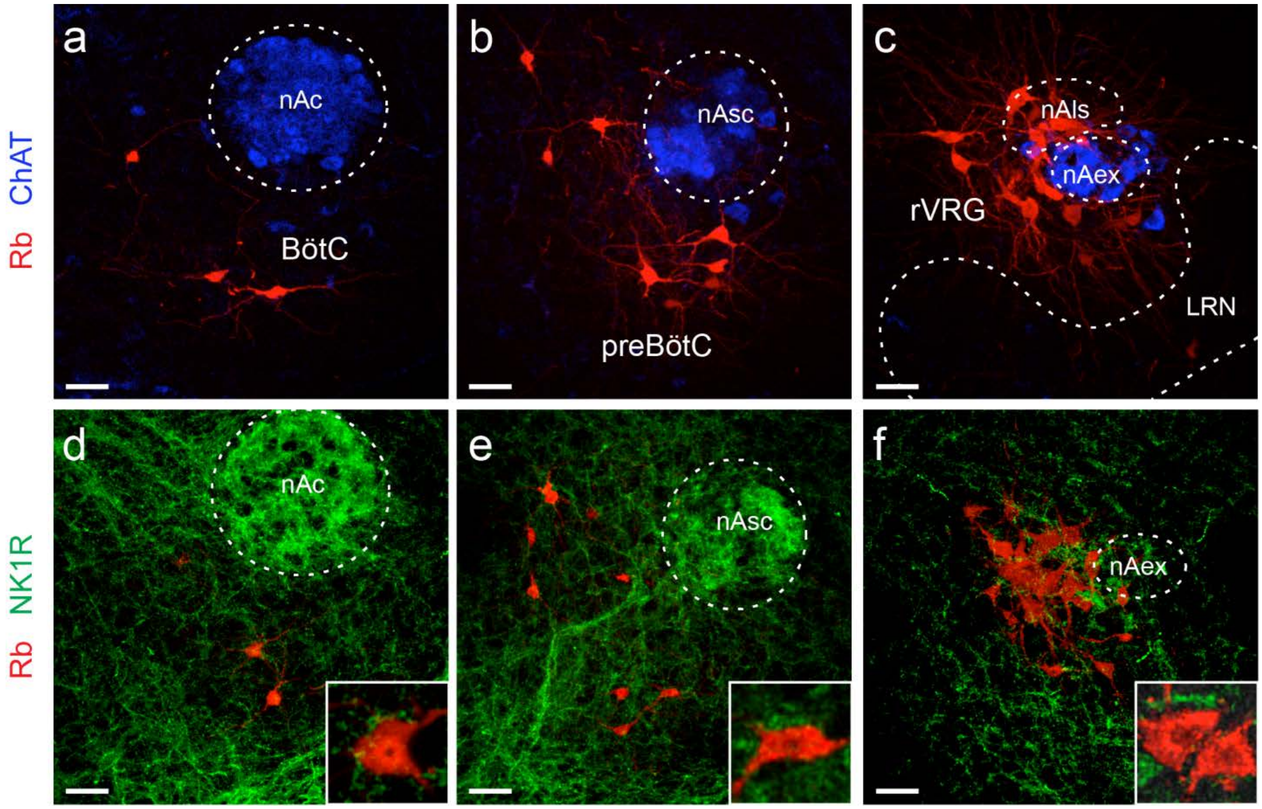

Supplementary Figure 4. The Ph-preMNs of the ventral respiratory column are not NK1R^{+}. **a-c**, Transverse sections showing the position of trace^{+} (red) Ph-preMNs in (**a**) the BötC located ventral to the compact formation of the nucleus ambiguus (nAc), (**b**) the preBötC ventral to the to the semi-compact formation of the nA (nAsc) and (**c**) the rVRG ventral to, and intermingled with, respectively the loose formation (nAls) and the external part (nAex) of the nA. **d-f**, Example image of the same regions showing NK1R immunostains (green) and trace^{+} Ph-preMNs immuno-negative for NK1R at low magnification and close up views. Scale bars: 50μm.

Supplementary Figure 5

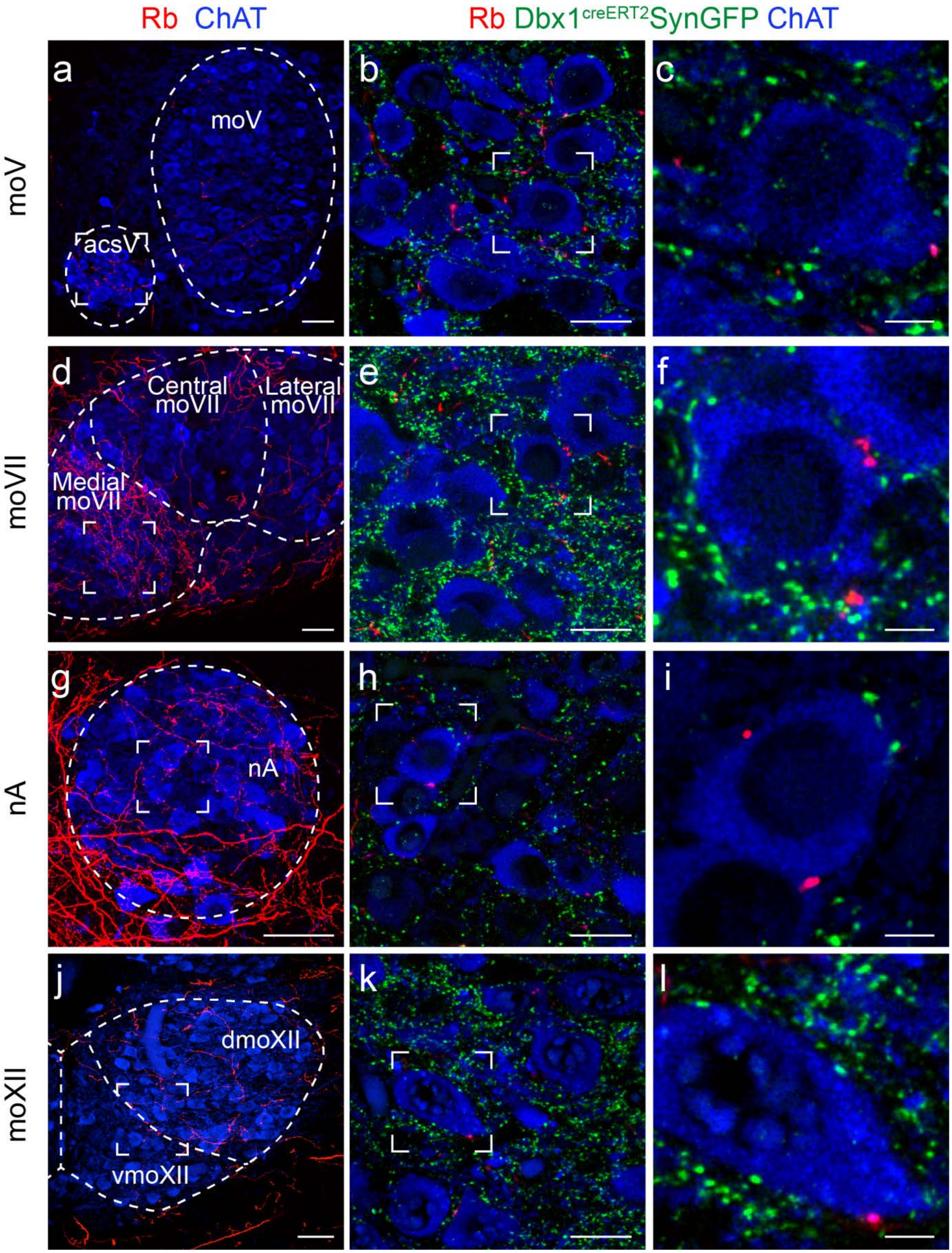

Supplementary Figure 5. V0 Ph-preMNs do not project on cranial motor nuclei. **a,** Trace^{+} (red) projections from Ph-preMNs onto ChAT^{+} (blue) trigeminal (moV) and accessory part of the trigeminal nucleus (acsV). **b**, Zoom of the square inset in a showing the presence of non-V0 Ph-preMNs projections (mCherry^{+} only, red) and V0 non-Ph-preMNs synaptic terminals (GFP^{+} only, green) but absent (0.12 ± 0.04 boutons/soma, n=67 cells) V0 Ph-preMNs double labeled synaptic terminals (mCherry^{+}/ GFP^{+}, yellow). **c**, Single optical section of the inset in b. **d-f**, Corresponding panels in the facial motor nucleus (moVII, 0.10 ± 0.04 boutons/soma, n=63 cells), (**g-i**) nucleus ambiguus (nA, 0.00 ± 0.00 boutons /soma, from 50 cells) and (**j-l**) hypoglossal motor nucleus (moXII, 0.06 ± 0.03 boutons/soma, n=50 cells). Scale bars: a,d,g,j, 50µm; b,e,h,k, 20µm; c,f,i,l, 5µm.

Supplementary Figure 6

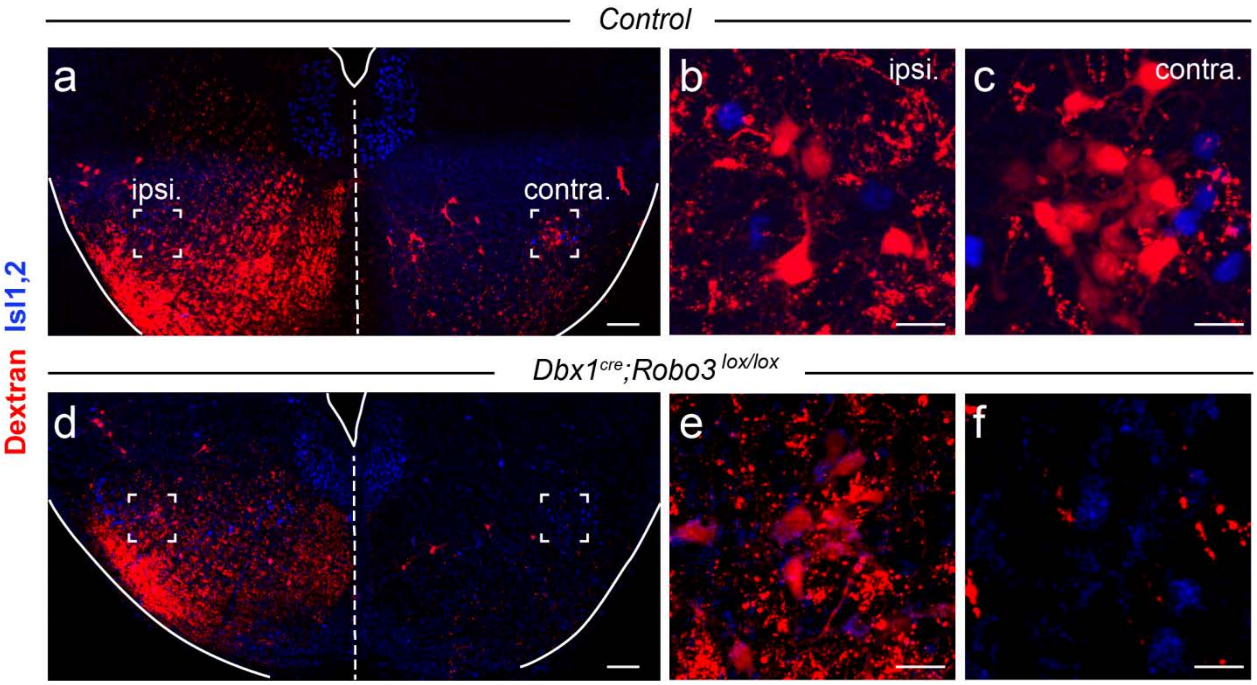

Supplementary Figure 6. Commissural axons of rVRG neurons are impaired in *Dbx1*^{cre};*Robo3*$^{lox/lox}$ mutants at E15.5. **a**, Transverse section at the level of the rVRG counterstained with Islet1,2 marking motor neurons after unilateral injection of rhodamine dextran dye in the phrenic motor column and showing bilaterally retrogradely labeled ispi- and contra-lateral rVRG neurons (insets) in control preparations. **b** and **c** are close-up views of the ipsi (**b**) and contra (**d**) insets in a. d, In *Dbx1*^{cre};*Robo3*$^{lox/lox}$ the tracer only labels the ipsilateral rVRG. **e** and **f** are close-up views of the ipsi (**e**) and contra (**f**) insets. Scale bars: a,d, 100µm; b,c,e,f, 20µm.

Supplementary Figure 7

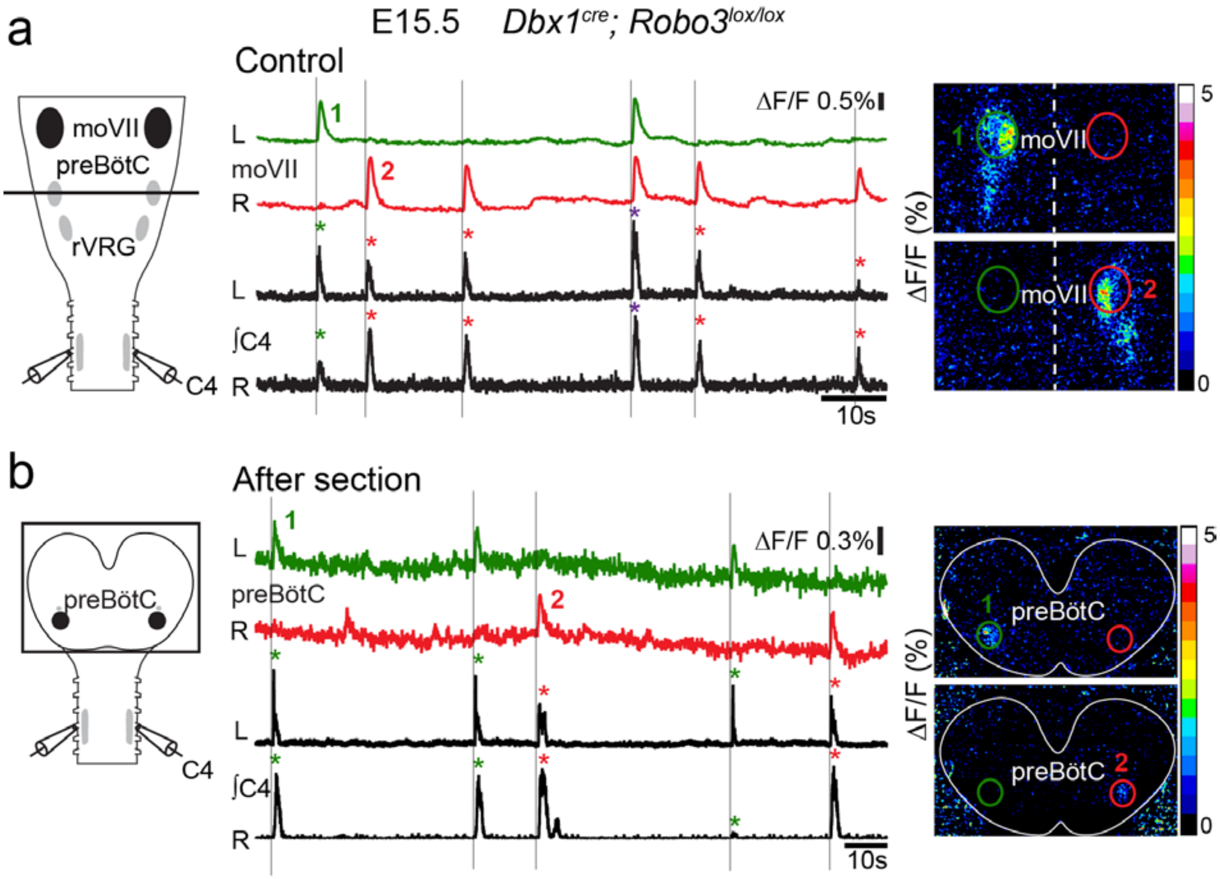

Supplementary Figure 7. Facial motor neurons (moVII) are ipsilaterally driven by the left and right decoupled preBötC in *Dbx1^{cre};Robo3$^{lox/lox}$* preparations. **a**, Left, schematic of the E15.5 brainstem-spinal cord preparation used to concurrently record control activities, of the left (L, green trace) and right (R, red trace) moVII by calcium imaging, and of the left and right C4 roots by electrophysiology. Generally independent occurrences of activity on the left (example peak 1) and the right (example peak 2) moVII (corresponding ΔF/F fluorescence changes illustrated in panels at right) are associated to synchronous (vertical bars) bilateral discharges of the left and right C4 roots. Note a tendency for C4 bursts ipsilateral to the active moVII to have larger amplitudes than the contralateral ones (green and red asterisks mark respective left active and right active moVII, purple asterisks indicate a rare event of synchronous left and right activation). **b**, Left, schematic of the preparation after a transverse section (black horizontal line on the schematic in a) exposing the preBötC for calcium imaging. Note that independent occurrence of activity on the left (example peak1) and right (example peak2) preBötC (corresponding ΔF/F fluorescence changes are illustrated in panels at right) also lead to bilateral synchronous unbalanced drives of C4 roots.
